# Not Only Protein: Dietary Supplements to Optimize the Skeletal Muscle Growth Response to Resistance Training: The Current State of Knowledge

**DOI:** 10.5114/jhk/18666

**Published:** 2024-04-15

**Authors:** Antonio Paoli, Giuseppe Cerullo, Antonino Bianco, Marco Neri, Federico Gennaro, Davide Charrier, Tatiana Moro

**Affiliations:** 1 Department of Biomedical Sciences, University of Padova, Padova, Italy.; 2 Department of Psychology, Educational Science and Human Movement, University of Palermo, Palermo, Italy.; 3 Italian Fitness Federation, Ravenna, Italy.

**Keywords:** muscle hypertrophy, strength training, ergogenic aids

## Abstract

Regarding skeletal muscle hypertrophy, resistance training and nutrition, the most often discussed and proposed supplements include proteins. Although, the correct amount, quality, and daily distribution of proteins is of paramount importance for skeletal muscle hypertrophy, there are many other nutritional supplements that can help and support the physiological response of skeletal muscle to resistance training in terms of muscle hypertrophy. A healthy muscle environment and a correct whole muscle metabolism response to the stress of training is a prerequisite for the increase in muscle protein synthesis and, therefore, muscle hypertrophy. In this review, we discuss the role of different nutritional supplements such as carbohydrates, vitamins, minerals, creatine, omega-3, polyphenols, and probiotics as a support and complementary factors to the main supplement i.e., protein. The different mechanisms are discussed in the light of recent evidence.

## Introduction

It is well known that proper nutrition is a fundamental complement to resistance training for the maintenance and growth of skeletal muscle mass. The main dietary components that affect muscle response to training are adequate caloric intake (Slater et al., 2019), the amount and quality of protein consumption and, less important, timing ingestion (Morton et al., 2018). A less investigated variable that supposedly may influence muscle response to training is the equilibrium of the “milieu interieur” or, to better say, a healthy muscle environment. A well-fitting example is inflammation: whilst acute inflammation plays a role in muscle adaptation to training, chronic inflammation may lead, instead, to muscle atrophy. The aim of this review is to provide an overview of the principal dietary supplements that can positively influence muscle response to resistance training.

### A Brief Overview of the Muscle Hypertrophy’s Mechanisms Induced by Resistance Training

Resistance training induces skeletal muscle hypertrophy via a sequential cascade of acute events:

1. activation of muscle fiber contraction;
2. signaling pathway activation resulting from:
  - mechanical stress of the muscle fibers nuclei and membrane,
  - release of systemic hormones,
  - release of myokines involved in the immune and acute inflammatory response;
3. increased muscle protein synthesis (MPS) through the activation of protein transcription and translation (Lim et al., 2022).

Moreover, the increased ribosomal biogenesis and activity, the proliferation of satellite cells and myonuclear accretion facilitate the increase in the muscle fiber cross-sectional area. The main actor of the skeletal muscle hypertrophy signaling cascade is the mammalian/mechanistic target of rapamycin complex 1 (mTORC1). Activation of mTORC1 signaling induces translation initiation, elongation, and a net increase in protein synthesis. In skeletal muscle, mTORC1 activity is stimulated not only by muscle contraction, but also by insulin and other nutrients such as amino acids (AAs) in general and Leucine in particular. The crucial role of mTORC1 in muscle protein synthesis stimulation has been shown in two different studies, during which rapamycin (a specific inhibitor of the mechanistic target of rapamycin, mTOR) was administered prior to ingesting essential AAs or performing a bout of resistance training exercises; the inhibition of mTORC1 led to a blunted response of muscle protein synthesis (Dickinson et al., 2011; Drummond et al., 2010; Gholami et al., 2022; Korczak et al., 2016). These findings indicate that mTORC1 activation is required either for AAs or exercise-induced increases in protein synthesis in humans, and that the load-induced activation of mTORC1 is a critical component for muscle growth. As previously stated, mTORC1 is influenced by nutrition and, mainly, by protein quantity and quality. That is, to stimulate and promote muscle hypertrophy, individuals involved in strength training programs or aiming to increase muscle mass need higher protein intake to trigger the abovementioned mechanisms. Nevertheless, proteins do not represent the sole supplement needed for such a purpose.

### Proteins

Although the focus of this review is on dietary supplements other than proteins, it is important to briefly mention the latter in the context of resistance training induced hypertrophy. On the one hand, proteins represent the most powerful nutritional stimuli to promote muscle protein synthesis, on the other, they provide muscles with amino acids, which are the fundamental building blocks for tissue construction. During exercise, and especially during resistance training, muscles undergo mechanical stress that temporarily increases the rate of protein breakdown (MPB) (Biolo et al., 1995), thus, proper AA availability is an important tool to guarantee muscle response to exercise. An efficient protein integration considers the quality, the quantity, and the timing of its ingestion.

#### Protein Quantity

Indications from FAO/WHO/UNU (2007) suggested an average protein intake requirement of 0.66 g/kg/d (Joint WHO/FAO/UNU Expert Consultation, 2007), whereas the recommended dietary allowance (RDA) (Trumbo et al., 2002) and the population reference intake (PRI) (EFSA, 2017) indicate a daily level of protein intake of 0.83 g/kg. The latter is considered sufficient to meet the nutrient requirements of nearly 97.5% of healthy people. Normally, daily protein turnover is around 250 g/day, but this value may be higher in athletes and should be calculated in consideration of the total energy intake. In 2002, the Institute of Medicine introduced the concept of Acceptable Macronutrient Distribution Range (AMDR). AMDR suggests instead that protein intake should provide 10–35% of total energy intake (Wolfe et al., 2017), which corresponds to 0.8–2.4 g/kg. Accordingly, some authors believe that protein AMDR of 10–35% may be more appropriate than PRI or RDA to fit different needs (including athletes) (Phillips, 2017). Indeed, available data suggest that a strength/power/body building athlete needs at least 1.7 g/kg of protein (Bandegan et al., 2019) during non-training days to guarantee a proper muscle hypertrophic response. Moreover, even the need of proteins to support muscle mass remodeling for an endurance athlete may be higher (1.6–1.83 g/kg) than it was commonly thought, being even higher the day following a training session (Bandegan et al., 2019).

#### Amount of Protein per Meal

Studies are few and contradictory, especially when exercise is included in the experimental design. For example, Witard and colleagues (2014) observed a plateau effect at 20 g per meal, suggesting that increasing protein intake beyond that threshold does not provide further advantage. Conversely, other authors showed that 40 g of protein per meal can promote higher protein synthesis compared to the 20-g amount. This discrepancy could be dependent on some variables of the training intervention, such as only quadriceps femoris muscle (i.e., leg extension) being involved in the first study versus a total body training program in the second one. Consequently, for a total body training session (or a session that involves many large muscles) the maximal protein intake may be set around 40 g per meal. Very recently, a study by Trommelen and colleagues (2023) showed that the ingestion of 100 g of protein resulted in a greater and more prolonged (>12 h) anabolic response when compared to the ingestion of 25 g of protein. These findings suggest that the anabolic response to protein ingestion has probably no upper limit as previously reported in vivo humans (Trommelen et al., 2023).

#### Quality of Protein

The more recent index of the quality of the protein source is the Protein Digestibility-Corrected Amino Acid Score (PDCAAS), whilst the most reliable method to assess the quality of a protein seems the Digestibile Indispensabile Amino Acid Score (DIAAS) (Table 1). In the DIAAS, the quality of protein is based on the relative digestibility composition of essential amino acids (IAAS) and amino acid necessity.

**Table 1:** PDCAAS and DIAAS values. Limited AA calculated with an AA reference ratio according to different proteins sources.

Protein source	PDCAAS	DIAAS	Limiting AA
Whey protein isolated	1.00	1.09	His
Whey protein concentrate	1.00	1.18	Met+Cys
Soy protein concentrate (mean of two sources)	0.989	0.902	Met+Cys
Pea protein concentrate	0.893	0.822	Met+Cys
Rice protein concentrate	0.419	0.371	Lys
Cooked kidney beans	0.648	0.588	Met+Cys
Cooked rice	0.616	0.595	Lys

*PDCAAS: Protein Digestibility-Corrected Amino Acid Score; DIAAS: Digestibile Indispensabile Amino Acid Score; AA: amino-acids*

Opposite to the PDCAAS, the DIAAS is not based on a single reference protein. For this reason, the DIAAS can classify all the reference proteins based on their theoretical quality (blend with specific properties too). The DIAAS can be calculated as follow: DIAAS (%) = 100 x [(mg of digestible dietary IAA in 1 g of the dietary test protein)/(mg of the same amino acid in 1 g of the reference protein)] (Rutherfurd et al., 2015).

**Figure 1:**
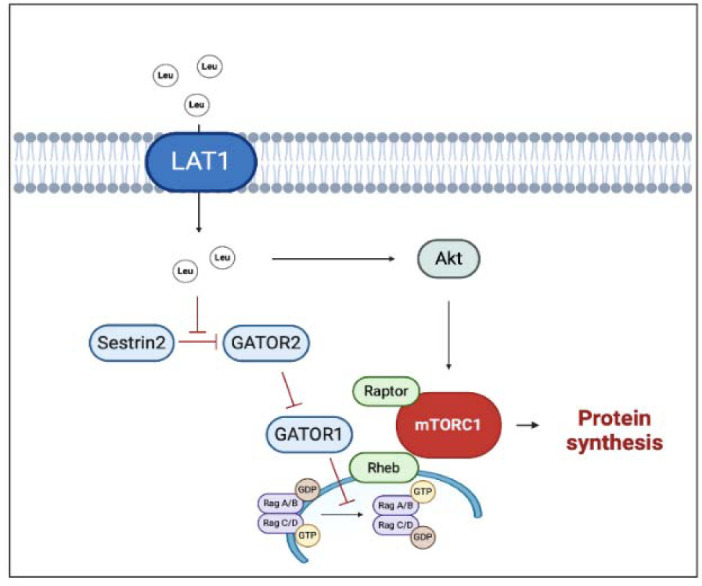
Multiple effects of leucine on hypertrophic response. The black arrows suggest the main effects driven by leucine on anabolic factors (i.e., improved protein synthesis). The truncated red arrows indicate inhibitory activity. AMPK, AMP activated protein kinase; GATOR1/2, Gap activity toward Rags 1/2; GDP, Guanosine-5'-diphosphate; GTP, Guanosine-5'-triphosphate; LAT1, L-type large neutral amino acid transporter small subunit 1; Leu, Leucine; mTORC1, mammalian/mechanistic target of rapamycin complex 1; Rheb, Ras homolog enriched in brain. Created with BioRender.com

Regarding amino acid composition, a pivotal role is played by a branched-chain amino acid (BCAA) and specifically by the one, i.e., leucine, which can directly stimulate muscle protein synthesis via mTORC1 signaling activation. To stimulate protein synthesis, mTORC1 needs to move close to the lysosome membrane, where the interaction with the Ras homolog enriched in brain (Rheb) allows mTORC1 activation (Moro and Paoli, 2020). BCAAs benefit from specific transporters, such as the sodium-independent L-type large neutral amino acid transporter small subunit 1 (LAT1), that can directly or indirectly transport BCAAs inside the cell. Once inside the cytosol, leucine is perceived by a specific sensor (Sestrin 2) and blocks its inhibitory effect on a series of protein complex (GATOR 1 and GATOR 2) enhancing mTORC1 activation (Moro et al., 2016) (Figure 1). Whilst the daily amount of leucine seems to influence the whole muscle protein metabolism, the leucine meal distribution appears to influence all the above-described mechanisms. Leucine supplementation was able to restore MPS even with a suboptimal amount of meal protein. Indeed, a large body of evidence suggests a threshold for the anabolic effect of leucine, which has been set to around 2.5 g of leucine within a single meal (Borack and Volpi, 2016; Fry et al., 2011). In practical terms, an athlete devoted to muscle hypertrophy needs, at least, 30 grams of high-quality protein per meal. The above calculation must be applied to a good quality protein in which the amount of leucine is relatively high (≈ 2.5 g of leucine).

### Carbohydrates

The role of carbohydrates (CHO) in resistance training and muscle hypertrophy is still not fully understood. As a matter of fact, most of the research focuses almost entirely on CHO intake for endurance training, while the specific recommendations for resistance training have yet to be defined (Rodriguez et al., 2009). As general recommendations, the American College of Sports Medicine, the American Dietetic Association, and the Dieticians of Canada recommend CHO targets ranging from 3 to 5 g/kg/day for low-intensity or skill-based activities to 8–12 g/kg/day for very high training demands. Similarly, intake of 3–5 g/kg/day for strength and power athletes and 3–7.2 g/kg/day for bodybuilders has been reported. It should be noticed that, even though moderate intake of CHO has been recommended, some studies suggest that lower intake of CHO does not impair both performance and post exercise cellular processes, such as MPS.

As we mentioned before, during resistance training different signaling pathways trigger a cascade of cellular responses that lead to muscle growth. Carbohydrate ingestion induces an increase in insulin and insulin growth factor 1 (IGF-1) concentration, which are potent anabolic molecules and can directly stimulate phosphatidylinositol 3-kinase (PI3K), a protein kinase-B (AKT) pathway. The activation of AKT induces the phosphorylation of the tuberous sclerosis complex (TSC) and its dissociation from Rheb, allowing Rheb to associate with mTORC1 and, in turn, stimulate the synthesis of new proteins (Schiaffino and Mammucari, 2011).

**Figure 2:**
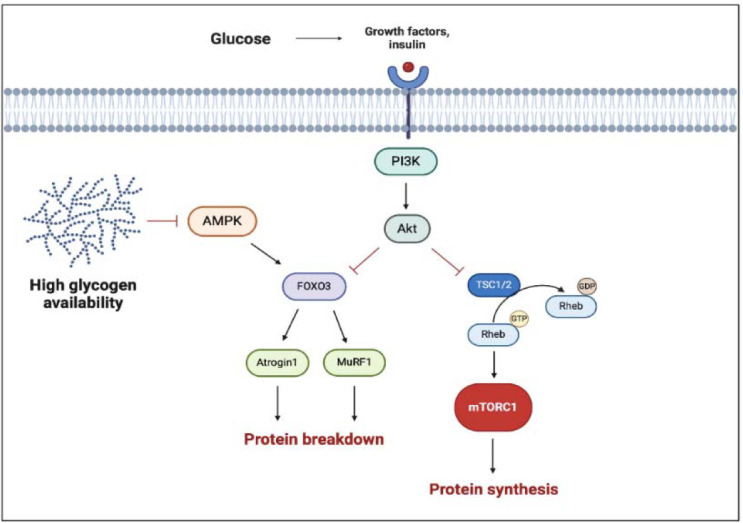
Multiple effects of carbohydrates and glycogen on hypertrophic response. The black arrows suggest the main effects on anabolic factors (i.e., enhanced proteinsynthesis). The truncated red arrows indicate inhibitory activity (i.e., protein breakdown). AMPK, AMP activated protein kinase; FOXO3, Forkhead box O3; mTORC1, mammalian/mechanistic target of rapamycin complex 1; MuRF-1, muscle ring finger-1; PI3K, phosphatidylinositol 3-kinase; Rheb, Ras homolog enriched in brain; TSC1/2, tuberous sclerosis complex. Created with BioRender.com

On the other hand, carbohydrate ingestion inhibits AMP-activated protein kinase (AMPK), an energetic state sensor, which regulates the FoxO-dependent protein degradation pathways and has an inhibitory effect on mTORC1. AMPK can activate FoxO factors by phosphorylation at several regulatory sites distinct from AKT phosphorylation sites (Schiaffino and Mammucari, 2011). The AMPK activation, due to energetic depletion, permits to reactivate ATP storage with catabolic processes, such as glycogenolysis and FFA mobilization. Lastly, AMPK increases the mitochondrial FFA up-take in skeletal muscles (Figure 2). As reported by Trommelen and colleagues (2015), the infusion of exogenous insulin efficiently enhances muscle protein synthesis, but this effect is clear only with the concomitant introduction of amino acids (Trommelen et al., 2015). Furthermore, other authors have reported no additional effects on MPS by combining protein and CHO (Glynn et al., 2010). These results suggest that insulin has no direct effect on MPS, despite being permissive for it. On the other hand, insulin can affect muscle growth by inhibiting muscle protein breakdown (via AKT inhibition of the FoxO3 pathway), thereby fostering a positive protein balance.

Carbohydrates also play a crucial role in guaranteeing glycogen availability, which is involved in the regulation of the anabolic response to resistance training (Box 1). In fact, the primary precursor for glycogen synthesis is represented by glucose, derived from newly ingested carbohydrate, or gluconeogenic precursors such as like lactate or alanine. Daily CHO intake of ~7–10 g/kg body mass is necessary to totally fill the glycogen storage. Glycogen synthesis is more effective with dietary CHO sources that elicit higher blood glucose (a high glycaemic index, GI) and insulin responses. As showed by Burke and colleagues (2017), glycogen storage increases during 24 h of recovery after CHO-rich meals based on high-GI food compared with an identical amount of CHO ingested in a form of low-GI food (Burke et al., 2017).

### Omega 3

The n-3 polyunsaturated fatty acids (omega-3), eicosapentaenoic acid (EPA) and docosahexaenoic acid (DHA) are present in oily fish and their effects on cardiovascular health and inflammation have been widely investigated. However, their effects on muscle anabolism are still poorly understood. EPA and DHA inhibit many aspects of inflammatory processes such as leucocyte chemotaxis, production of eicosanoids like prostaglandins and leukotrienes and the production of pro-inflammatory cytokines. Omega-3 also increases the production of pro-resolution molecules: resolvins, protectins and maresins that stimulate self-limited innate responses, enhance innate microbial killing, and protect the organism. Moreover omega-3 down-regulates the expression of inflammatory cytokine genes (via NFκB, PPAR-γ, GPR120 etc.) and acts on intracellular signaling (via increased content of EPA and DHA in specific membrane regions) (Calder, 2015).

It must be underlined that inflammation is a double-edged sword for muscle mass growth. On the one hand, it has been suggested that inflammation is essential for muscle response to exercise whilst, on the other hand, a chronic inflammation has been linked to negative effects on muscle mass (Paoli et al., 2015) and training related responses. The overload induced by physical exercise may cause different degrees of microtraumas in skeletal muscles related to the subsequent tissue regeneration process. Indeed, this regeneration process is strictly related to the local inflammation evolution (e.g., evolution from macrophages type 1 to type 2) and also involves satellite cell activation (Walton et al., 2019). The bottom line is that, whilst a chronic low grade inflammatory status is deleterious, the acute inflammatory response to exercise seems to be fundamental to stimulate the muscle repair process and satellite cell proliferation.

**Figure 3:**
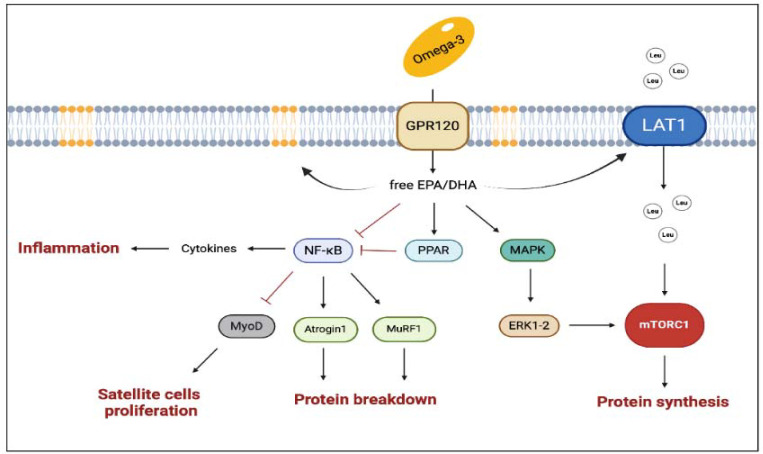
The main pathways involved in the multiple effects of omega-3 on muscle health. The black arrows suggest the main effects influenced by enhanced concentration of EPA and DHA (i.e., enrichment of EPA and DHA into membrane phospholipids, enhanced protein synthesis). The truncated arrows refer to the mechanisms inhibited by omega-3 (i.e., protein breakdown, inflammation, decline in satellite cell proliferation, and improved mitochondrial dynamics). DHA, docosahexaenoic acid; EPA, eicosapentaenoic acid; GPR120, G-coupled protein receptor 120; LAT1, L-type large neutral amino acid transporter small subunit 1; MAPK, Mitogen-Activated Protein Kinase; mTORC1, mammalian target of rapamycin complex 1; MuRF-1, muscle ring finger-1; MyoD, myoblast determination protein 1; NF-κB, nuclear factor kappa-light-chain-enhancer of activated B cells; PPARs, peroxisome proliferator-activated receptors. Created with BioRender.com

Accordingly, omega-3 can regulate the inflammatory process and potentially optimize the skeletal muscle response to training. Omega-3 can indeed change the lipid composition of cell membranes, and therefore alter their affinity with the lipid rafts and promote their action in the intracellular signaling pathway. Within this framework, omega-3 increases peroxisome proliferator-activated receptors (PPARs) expression as PPARs interfere with the translocation to the nucleus of the NFκB (Polus et al., 2016). If inhibited, NFκB cannot act on the ubiquitination pathway, and, in turn, the protein breakdown derivate from the muscle ring finger-1 (MuRF-1) is attenuated (Figure 3). However, the effects of omega-3 on muscle growth are not totally depended on their anti-inflammatory action. It has been suggested that a diet rich in omega-3 could enhance mTor activation by increasing the availability of amino acids. This hypothesis has been corroborated, for example, by showing an increase in the L-type amino acid transporter 1 (LAT1) expression (which is involved in the leucine transport within cells) during a diet rich in omega-3 (Dodd and Tee, 2012; Li et al., 2015). Other authors showed an increase in the mTORC1 signaling pathway without a direct activation of mTORC1 (Kamolrat and Gray, 2013). In vitro studies showed an interaction of DHA and EPA with the Mitogen-Activated Protein Kinase (MAPK) cascade, which can stimulate cell proliferation; moreover, EPA seems to increase MyoD and myogenin expression and to promote satellite cell differentiation. In summary, the available evidence suggests that omega-3 may support muscle growth, however, the exact mechanism by which they exert their positive effect is still unknown (McGlory et al., 2019).

### Micronutrients

#### Vitamins

Vitamins are essential compounds, required in a lower amount than macronutrients, and they are involved in many vital processes (e.g., neurological, energetic, and metabolic), fundamental from the cellular level to the whole organism.

Fat-soluble vitamins (A, D, E, and K) can be stored in various tissues, resulting in toxicity if consumed in excessive amounts. Otherwise, high levels of water-soluble vitamins (complex of B-vitamins and vitamin C) are generally eliminated in urine without significant harmful effects, with some exceptions (e.g., vitamin B6, where excessive intake is related to neurological damage). Basically, vitamin supplementation should not be necessary when following a balanced diet. In other cases, vitamins help avoid micronutrient deficiencies that might be detrimental for muscle health (Kerksick et al., 2018). For example, consumption of vitamin supplements with antioxidant properties (i.e., vitamins C and E) is also frequent. Reactive Oxygen Species (ROS) production is a physiological consequence of muscle activity, and it is involved in the regulation of skeletal muscle hypertrophy (Merry and Ristow, 2016). On the other hand, excessive oxidative stress can have negative effects on skeletal muscle contractile function (Merry and Ristow, 2016). Thus, supplementation with antioxidant vitamins (i.e., vitamins E and C) can play a role in protecting muscles from oxidative stress, improving exercise tolerance in athletes, and helping to maintain a healthy immune system during heavy training periods (Martinez-Ferran et al., 2020). A recent meta-analysis supported the role of vitamin C in reducing lipid peroxidation and IL-6 production in response to a single exercise training session (Righi et al., 2020). During physical inactivity, vitamin E supplementation might counteract muscle protein breakdown by reducing the expression of several factors responsible for increased proteolysis (i.e., calpain, caspase-3, -9 and -12 and MuRF1 and MAFbx). Indeed, vitamin E exerts a protective role in the treatment of sarcopenia by limiting catabolic processes that determine muscle loss (mainly type II fibres) (Chung et al., 2018). Contrastingly, ingesting high doses of vitamins C and E for long periods (1000 mg and 230 mg for 10 weeks) negatively interfered with the acute cellular response to high-intensity resistance training by blunting phosphorylation of p38 mitogen-activated protein kinase, extracellular signal-regulated protein kinases 1 and 2 and p70S6 kinase after the exercise session (Paulsen et al., 2014), likely contributing to attenuating hypertrophy stimuli over time (Dutra et al., 2020).

While vitamin D is a fat-soluble vitamin primary known for its role in bone metabolism, recent studies on myoblasts highlighted further involvement of vitamin D at the muscular level as well, specifically in the processes of differentiation, proliferation, and growth of muscle cells. Vitamin D participates in the modulation of muscle contraction and the expression of strength by controlling calcium homeostasis (Uchitomi et al., 2020). Interestingly, vitamin D influences muscle remodeling directly as well as indirectly. For instance, at the intracellular level, vitamin D can directly promote an anabolic effect by activating the ERK 1/2-p38 MAPK signaling pathway, through the steroid receptor coactivator complex (Src). Consistently, a deficiency in vitamin D has been associated with a direct expression increase in various factors (e.g., atrogin-1 and MuRF1) that leads to muscle atrophy (Dawson-Hughes, 2017). Vitamin D affects indirectly protein synthesis by conditioning the availability of amino acids, regulating the activity of transporters at the renal and intestinal levels, and by reducing oxidative stress (Dzik and Kaczor, 2019). At present, there is still a debate about the effects of vitamin D supplementation on muscle growth and strength. It seems that, combined with resistance training, vitamin D supplementation (2000 U.I. daily for 16 weeks) could influence muscle remodeling by improving the fiber type 2 percentage. Nevertheless, this does not lead to a greater increase in hypertrophy when compared to exercise alone (Agergaard et al., 2015). Similarly, vitamin D supplementation (8000 IU daily for 12 weeks) eliminates vitamin D deficiency without influencing muscle growth in young vitamin D-deficient men (Savolainen et al., 2021).

A systematic review and meta-analysis, including 30 randomized controlled trials assessing the effects of vitamin D supplementation on muscle function, concluded that vitamin D supplementation had no impact on muscle gain or muscle strength (Beaudart et al., 2014). However, another systematic review and meta-analysis, including trials that combined vitamin D supplementation with resistance training interventions, found an improvement in muscle strength in elderly subjects taking vitamin D supplementation compared with exercise only (Antoniak and Greig, 2017). Overall, vitamin D may enhance muscular response to resistance exercise. However, further investigations are necessary to identify the best protocol for taking vitamin D, including the appropriate dosage and duration.

In general, when athletes fail to achieve their energetic needs and subsequently to consume adequate micronutrient intake, a low-dose daily multivitamin and/or a vitamin supplement might be considered (Iraki et al., 2019). A higher dosage of some vitamins, such as vitamins C and E, could be proposed during short periods of high-intensity training to reduce muscle damage (Martinez-Ferran et al., 2020) considering the blunting effects of higher doses of antioxidants on muscle hypertrophic response.

#### Minerals

Minerals are vital inorganic elements required for numerous metabolic processes. They provide structural support for tissues, play crucial roles in enzyme and hormone functions, and help regulate metabolic and neurological processes. Poor mineral levels may hamper exercise capacity, whereas supplementing minerals in deficient athletes has been found to enhance exercise capacity (Kerksick et al., 2018). Some minerals such as magnesium, zinc and chromium, are particularly related to the muscle function and the remodeling process. For instance, magnesium seems to contribute to muscle contraction and relaxation activity. However, it has also been suggested that magnesium might improve muscle mass/strength owing to its influence on MPS. As reported for the first time in 2021, magnesium supplementation can enhance myoblast differentiation and protein synthesis by activating mTOR signalling in aged mice (Liu et al., 2021).

Similar effects of dietary magnesium intake in relation to muscle strength and mass have been observed in humans as well. Indeed, serum magnesium is significantly lower in sarcopenic subjects compared with nonsarcopenic adults (ter Borg et al., 2016). Moreover, subjects with deficiency of magnesium (e.g., older adults and alcoholics) may benefit when supplementing this inorganic element rather than active people with a good magnesium status (Wang et al., 2017). However, evidence regarding magnesium supplementation and muscle health remains scarce.

In a similar fashion, the zinc intake has been linked to greater muscle mass in adults. Zinc is a cofactor for over two hundred enzymes in the human body and stored mainly in muscle mass. In muscle tissue, where more than 50% of body zinc is stored, zinc contributes to the control of muscle mass in multiple ways. For instance, as observed in an in-vitro study, zinc plays a key role in promoting the proliferation of myoblasts, and in the differentiation and maturation of myofibers (Mnatsakanyan et al., 2018). Furthermore, zinc status has been related to serum testosterone levels in healthy adults and the supplementation of this inorganic element has been showed to raise testosterone levels in adults with zinc deficiency and to enhance muscle mass in children with growth failure (Vincent, 1999).

As deeply reviewed by Hernandez-Camacho and colleagues (2020), zinc seems to be involved in different proteostatic systems induced by exercise, such as autophagy, but further investigations are needed to clarify this aspect (Hernández-Camacho et al., 2020). For these reasons, zinc deficiency could negatively impact the protein turnover and muscle building as exercise adaptation in general. Having this in mind, there is no evidence that supports zinc consumption in a higher dosage than recommended dietary intake in order to obtain more benefits from training adaptation.

**Box 1**

The role of glycogen in resistance training is less well-defined compared to endurance training. Several studies have shown that muscle glycogen levels decrease by 30–40% following resistance training, but it is clear that duration, volume and intensity as well as the many other variables of RT are a source of great variability (Pascoe et al., 1993).

The depletion of muscle glycogen leads to a reduction in the rate of ATP replenishment, potentially contributing to fatigue by impairing sarcoplasmic reticulum calcium release (Ørtenblad et al., 2013). In more detail, it has been observed that Ca^2+^ handling is adversely affected when muscle glycogen is reduced, even when global ATP levels are constant (Ørtenblad and Nielsen, 2015). Therefore, glycogen decreases may indirectly contribute to a reduction in acute force output, and, in turn, to a decrease in the hypertrophic stimulus. Furthermore, mounting evidence hints toward a potential role of muscular glycogen not only in energy supply, but as a signaling molecule as well (Ørtenblad and Nielsen, 2015). Low glycogen availability (~160 mmol·kg-d.w.) can increase both resting and exercise-induced AMPK activation in humans, when compared to high levels of glycogen availability (~910 mmol·kg-d.w.). However, although it was shown that mTOR phosphorylation increased significantly with high glycogen availability, there were no noticeable changes in muscle protein synthesis (MPS), suggesting that small differences in signaling might have no effects on MPS (Camera et al., 2012). Additionally, glycogen may subserve the regulation of the hypertrophic response to exercise by influencing cellular osmotic pressure. For instance, several studies have shown that a high-carbohydrate diet leads to an increase in muscle glycogen, in parallel with a rise in body water content of approximately 2.7–4.0 grams per gram of glycogen, mostly due to intracellular water (Sherman et al., 1982). Cellular hydration, or cell swelling, acts as a physiological regulator of cellular activity. Despite fact that the exact physiological process connecting cell swelling to an anabolic drive is not completely understood, it is possible that the increased pressure on the cell membrane is interpreted as a danger to the cell's structure, prompting a signaling response to strengthen its ultrastructure. In addition, water bound to glycogen can ensure good availability of nutrients, optimizing energy resources and, as consequence, promoting anabolism. In summary, glycogen availability may play an important role in muscular response to exercise, but further evidence is still warranted to elucidate whether performing resistance training with low glycogen availability can bring to divergent skeletal muscle adaptations.

Chromium is another essential element in human health. Its role in nutrition is related to macronutrient metabolism mainly due to its insulinogenic function. Supplementation of chromium has been supposed to improve anabolic response to training sessions. In rats, the addition of chromium (2 g human equivalent dose) to BCAAs (6 g human equivalent dose) stimulates mTOR signaling proteins, and thus, significantly enhances MPS after training, compared to BCAAs alone (Komorowski et al., 2020). Post-exercise ingestion of chromium, combined with soy protein and amylopectin, has been shown to improve protein synthesis by reducing the phosphorylation of FOXO1, FOXO3 (regulators of ubiquitin-related proteolysis), and MAFbx, MuRF-1 (the main regulators of ubiquitin-related proteolysis) reducing muscle atrophy (Kayri et al., 2019). These mechanisms have been further confirmed by a recent study on the synergic action of chromium combined with different doses of whey protein, showing that the addition of chromium to whey protein results in a significant increase of 14.3% in MPS, compared to whey protein alone (Sahin et al., 2024). In a study with 154 adult subjects, aimed to investigate the impact of chromium supplementation (200 µg and 400 µg day) and its effects on body composition variables, without any additional training intervention, researchers found a significant decrease in body fat content (~1.4%) and an increase in free fat mass (~0.5 kg) in the supplemented group compared to the placebo (Kaats et al., 1996). Nevertheless, another study failed to show positive effects of chromium supplementation (200–800 μg/d for 4–16 weeks) on muscle growth (Campbell et al., 1999) even with exercise intervention (Campbell et al., 2002). At this stage, the current evidence of chromium supplementation for athletic performance is lacking, but further studies to explore the synergistic effect of chromium and aminoacids or proteins on muscle protein synthesis are needed.

### Polyphenols

Polyphenols are a category of molecules naturally found in plants and their products, including fruits, vegetables, tea, red wine, and chocolate. These compounds are widely recognized for their potential health benefits to many non-communicable diseases (NCD), aging, and immunity (Somerville et al., 2017). To date, over 8,000 polyphenols have been identified and classified into four main groups: flavonoids, stilbenes, lignans, and phenolic acids. Certain polyphenols, such as resveratrol, quercetin, and curcumin, have been linked to muscle health due to their strong antioxidant and anti-inflammatory effects. Oxidative stress and inflammation activate regulatory factors which induce protein catabolism and muscle atrophy.

For instance, resveratrol (3,5,4'-trihydroxy-trans-stilbene), a polyphenol primarily found in pines, berries, grape skins, and red wine, can stimulate anabolic pathways through enhancing Akt/mTOR signaling and attenuate proteolysis by downregulating the ubiquitin ligases atrogin-1 and MuRF-1, as observed in myotubes (Nikawa et al., 2021). Combined with exercise, resveratrol supplementation exhibits a synergistic effect on muscle adaptation. In aged rats, exercise with resveratrol supplementation (150 mg/kg/day) resulted in a higher cross-sectional area (CSA) compared with only exercise or resveratrol supplementation (Liao et al., 2017). Similarly, synergistic effects on strength and hypertrophy were reported when combining resveratrol with resistance training (Nikawa et al., 2021). In human subjects, resveratrol supplementation (500 mg/day) with physical exercise, including resistance training for 12 weeks, improved muscle adaptation leading to higher hypertrophy of type I and IIA muscle fiber sizes, and an increase in satellite cells and myonuclei in older adults compared with exercise training alone (Alway et al., 2017). However, although resveratrol has been recognized as a potential supplement to improve muscle adaptation, its mechanisms in human or animal models remain poorly understood, and new studies are needed to explore the synergistic effect of resveratrol supplementation on muscle remodeling with resistance training.

Similarly, quercetin (3,30,40,5,7-pentahydroxyflavone), a flavonoid naturally contained in apples, citrus fruits, and onions, appears to play a role in the muscle remodeling process. In models of induced muscle atrophy, quercetin prevented muscle loss *via* the reduction in several elements that regulate protein catabolism, such as NF-κB, ubiquitin ligases atrogin-1, and MuRF-1, while also sustaining muscle anabolism by increasing Akt phosphorylation (Otsuka et al., 2019). Beyond its therapeutic use, quercetin has been evaluated as an ergogenic aid, primarily in endurance training (Kerksick et al., 2018). For example, daily supplementation of 1 g of quercetin for 2 weeks, using a randomized, crossover design with a two-week washout period, improved 12-min treadmill time trial performance in untrained males (Nieman et al., 2010). Combined with resistance training, ingesting 1 g of quercetin 3 h prior to a single bout of resistance training seemed to improve neuromuscular performance during and after the training session (Patrizio et al., 2018). In the long term, quercetin supplementation (200 or 500 mg for 24 weeks), in addition to resistance training, enhanced passive muscle stiffness without improving muscle gain compared to resistance training alone (Otsuka et al., 2022). Despite promising findings in in vitro and in vivo models, quercetin supplementation studies are still at their early stages compared to other well-known muscle-building supplements (e.g., protein and creatine), and future investigations are necessary to understand the role of quercetin in optimizing muscle response to resistance training.

Curcumin (1,7-bis (4-hydroxy-3-methoxyphenyl) 1,6-heptadiene-3,5-dione), the main natural bioactive polyphenol of the spice herb turmeric (2%–5% by weight), is primarily known for its antioxidant and anti-inflammatory properties. Its antioxidant activity, about 10 times greater than vitamin E (M. Khopde et al., 1999), suppressed oxidative stress and increased myofibrillar proliferation, resulting in attenuated muscle loss in a mouse model of induced muscle atrophy (Nikawa et al., 2021). In humans, curcumin supplementation appears to reduce many biological markers of muscle damage and inflammation (CK, TNF-α, IL-6, and IL-8) following exercise-induced muscle damage. Findings from a systematic review suggested that curcumin ingestion, at a dose between 150 and 1500 mg/day, pre-, post- and during exercise, might improve performance and muscle recovery by attenuating exercise induced muscle damage and modulating the inflammation response caused by exercise (Fernández-Lázaro et al., 2020). However, to our knowledge, there is no research about the impact of curcumin supplementation on molecular mechanisms that govern muscle gains stimulated by resistance training.

**Figure 4:**
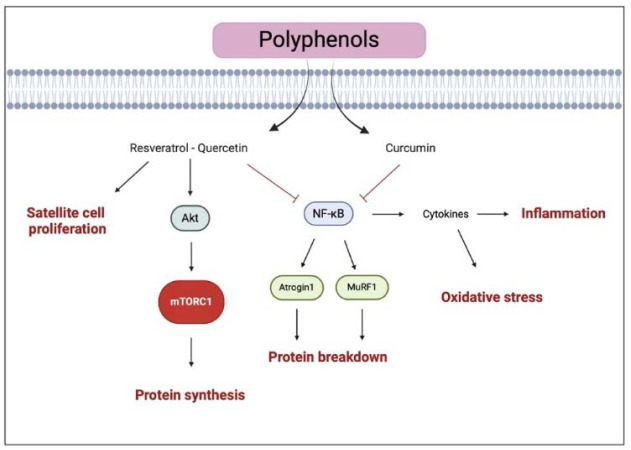
Illustration of the representative pathways of polyphenols effects on muscle health The black arrows suggest the main effects influenced by polyphenols (i.e., enhanced protein synthesis and satellite cell proliferation). The truncated arrows refer to the mechanisms inhibited by polyphenols (i.e., protein breakdown, inflammation, and oxidative stress). mTORC1, mammalian target of rapamycin complex 1; MuRF-1, muscle ring finger-1; NF-κB, nuclear factor kappa-lightchain-enhancer of activated B cells. Created with BioRender.com

In summary, polyphenol ingestion seems to promote muscle response to resistance training by enhancing antioxidant capacity and myofibrillar protein synthesis (Figure 4). However, the molecular mechanisms are still not fully understood, and further studies are needed to investigate the role of polyphenols in muscle gains.

### Creatine

Creatine (Cr), in particular creatine monohydrate (CM), is one of the most studied supplements, mainly for its effects on athletic performance. Creatine supplementation may be used in different sports involving repeated high-intensity exercise or different training programs such as resistance training or interval training (Kerksick et al., 2018).

The majority of creatine is stored within the skeletal muscle in the phosphorylated form of the high-energy product phosphocreatine (PCr); indeed, the pleiotropic effects of Cr depend particularly on the enzymatic reaction driven by creatine kinase (CK) and PCr intramuscular levels.

The system CK/PCr provides free energy for cell metabolism and a temporal energy buffer in cells of high energy requirement such as human skeletal tissues. Larger muscle creatine storage may enhance, by increasing the rate of PCr resynthesis, short term, high intensity exercise capacity to perform repeated bouts of maximal voluntary exercise (Maughan et al., 2018).

Kilduff et al. (2002) demonstrated that after five days of supplementation with Cr, fat-free body mass, peak force, and total force during a repeated maximal isometric bench-press test were greater in the group supplemented with creatine compared to the placebo (Kilduff et al., 2002).

Since supplementation increases the muscle creatine stores and PCr by near 20–40%, it is clear that adequate creatine loading leads to greater gains in lean mass, increases in isometric strength and enhancement in the acute performance of single and repeated bouts of high-intensity exercise (Maughan et al., 2018).

The updated position statements of the International Society of Sport Nutrition (ISSN) and the IOC consensus statement suggest that the most effective strategy for increasing creatine stores is the CM supplementation split into different phases (Kerksick et al., 2018; Maughan et al., 2018). The first phase is the so called “loading phase” in which 20.9 ± 4.5 g/day should be divided into four equal daily doses (5 g/dose or ~0.3 g/kg body weight) for 5–7 consecutive days. The following phase of the protocol requires a “maintenance phase” in which 3–5 g of CM should be provided for the entire duration of the supplementation period. It is interesting to note that the co-ingestion of carbohydrates and proteins (~50 g of proteins and CHO) increases the intramuscular creatine retention via an insulin mediated effect.

A second protocol involves the ingestion of 3 g/day of CM for 28 days. Even though this approach shows lesser side effects (i.e., gastrointestinal distress), it has lower effects on exercise performance (at least until creatine storage reaches full saturation).

No negative health effects were observed with long-term use (around 4 years of consequent supplementation) when either of these protocols was correctly employed (Maughan et al., 2018).

It is worth to underline that creatine supplementation may also be useful to help athletes during recovery periods, by accelerating recovery from injuries or from particularly intense training. It has been reported that creatine loading together with CHO supplementation provided before exercise is able to promote a greater glycogen restoration than carbohydrate alone (Kreider et al., 2017).

In general, there are available data suggesting that creatine exerts many positive effects on muscle growth through satellite cell activation, myonuclei number accretion, and increased protein synthesis via the IGF-1/4EBP1 pathway. Finally, another suggested hypothesis is represented by the enhanced vasodilation that may increase myocyte swelling and, in turn, muscle hypertrophy (Kreider et al., 2017).

### Prebiotics and Probiotics

Prebiotics are non-digestible substances that can selectively stimulate the activity and/or growth of colon bacteria, with a positive effect on the host’s health (Hill et al., 2014). Commonly used prebiotics include fructo-oligosaccharides (FOS), galacto-oligosaccharides, lactulose, and large polysaccharides (inulin, resistant starches). Instead, probiotics are bacteria and microorganisms present in certain food able to improve several health outcomes, such as microbiota composition, related with the human’s health and athlete’s performance (Di Dio et al., 2022). Indeed, it has been suggested that the improvement in intestinal barrier function and nutrient absorption may indirectly contribute to enhanced performance. For example, one study found that a twice daily probiotic supplementation of Bacillus coagulans GBI-30, 6086 (BC30) in combination with 20 g of casein increased vertical jump power in resistance trained participants compared to the control group, who consumed 20 g of casein alone (Georges et al., 2014). Authors suggested that improvement in the vertical jump might be related to enhanced muscle recovery related to gut microbial modulation. The enzymes produced by *B*. coagulans have been shown to aid the digestion of protein and carbohydrates and their addition to milk protein can enhance the rates of digested milk protein available for absorption. Moreover, a follow-up study demonstrated that the co-administration of *B*. coagulans GBI-30, 6086 with 20 g of casein provided positive effects on muscle damage and muscle soreness reduction as well as perceived recovery (Jäger et al., 2016). These benefits may allow for a better and faster adaptation to exercise training and, therefore, a faster increase in hypertrophy and performance. Since probiotics can improve digestive and immune health, enhance the gut intestinal barrier and the consequent nutrient absorption (Hill et al., 2014), accurate supplementation may improve the overall health of athletes and maximize the health benefits of protein supplementation (Jäger et al., 2016).

Interestingly, a probiotic supplementation of Bacillus subtilis DE111 with a post-workout recovery drink (composed of 45 g of carbohydrates, 20 g of protein, and 2 g of fat) reduced body fat and increased free fat mass compared to the placebo group. The authors supported the idea that probiotics, by the improvement of amino acid uptake and availability, contributed to a better body composition by increasing dietary protein-induced thermogenesis and altering satiety signaling (Townsend et al., 2018). However, most studies investigated the effects of probiotic supplementation and aerobic exercise performance, while less studies investigated the effects of probiotics in resistance training programs (Georges et al., 2014). As a matter of fact, a direct effect on muscle growth of probiotic supplementation has been demonstrated only in animal studies, using the L. plantarum TWK10 strain in mice (Chen et al., 2016). Jäger et al. (2019) raised a hypothesis that the anti-inflammatory effect of probiotics might implement the inhibition of protein breakdown, and, thus, might augment muscle mass; another hypothesis hinted toward the positive effect of probiotic administration on amino acid adsorption from protein (Jäger et al., 2016). However, more studies, especially in humans are required to better define the role and the potential mechanism of action of probiotics on muscle protein synthesis.

### Phosphatidic Acid

Phosphatidic acid (PA) is a diacyl-glycerophospholipid in which two fatty acids and a phosphate group are covalently bonded to a glycerol molecule through ester linkages. It serves as a signaling lipid, acts as a precursor for the synthesis of other lipids, and is a key component of cell membranes. Phosphatic acid levels are determined by endogenous synthesis (which is mainly prompted by mechanical stimuli), food introduction (small amount) and supplementation (Bond, 2017). Research has highlighted its potential role in muscle growth and performance enhancement. Through molecular-level investigations, it has been determined that PA exerts its effects by directly interacting with mTOR. PA's enhancing effect involves competitive inhibition by displacing FKBP38, an endogenous mTORC1 inhibitor, as well as allosteric activation of mTORC1 (Fang et al., 2001; Yoon et al., 2011). Additionally, it has been observed that PA supplementation can decrease muscle protein breakdown by reducing atrophy-related genes, which are upregulated in muscle protein catabolism (Shad et al., 2015). In rodent models, ingestion of PA resulted in elevated muscle protein synthesis (MPS) three hours after feeding, with or without concurrent whey protein supplementation (Mobley et al., 2015). These findings suggest that exogenous PA supplementation may increase mTOR-mediated signaling. However, human studies examining the effects of PA supplementation on muscle mass have led to controversial results. A study conducted by Hoffman et al. (2012) found that supplementing with 750 mg of PA showed potential benefits in increasing strength (1-RM bench press and 1-RM squat), as well as improving lean body mass in resistance-trained young adults. Accordingly, another study showed that supplementation with a multi-ingredient supplement containing 750 mg of PA enhanced lean body mass and the 1 RM bench press and squat in strength-trained men (Escalante et al., 2016). Conversely, another study found that PA supplementation, in combination with a 3 days•week^−1^ resistance-training program for 8 weeks, did not have a differential effect on changes in muscle thickness or 1RM strength compared with the placebo in resistance-trained participants (Gonzalez et al., 2017). Although some findings appear promising, a recent review emphasized the necessity of further investigations to fully comprehend the impact of PA supplementation on muscle growth and performance in humans (Teixeira et al., 2022).

## Conclusions

Proteins remain, so far, the most efficient dietary supplement to improve muscle mass. However, other dietary supplements can play a complementary role in sport nutrition and support the hypertrophic response to resistance training. Athletes seeking muscle growth should be sure to satisfy their daily intake of carbohydrates, omega 3 and other micronutrients. Under some conditions, a higher dosage of antioxidants should be considered to reduce muscle damage and improve recovery without affecting muscle gains. Further investigations about the role of other metabolites (such as phosphatidic acid) and bioactive compounds (including polyphenols, probiotics, and nitrates) in mechanisms that govern muscle response to resistance training are needed.
